# Relaxation of Natural Selection in the Evolution of the Giant Lungfish Genomes

**DOI:** 10.1093/molbev/msad193

**Published:** 2023-09-06

**Authors:** Silvia Fuselli, Samuele Greco, Roberto Biello, Sergio Palmitessa, Marta Lago, Corrado Meneghetti, Carmel McDougall, Emiliano Trucchi, Omar Rota Stabelli, Assunta Maria Biscotti, Daniel J Schmidt, David T Roberts, Thomas Espinoza, Jane Margaret Hughes, Lino Ometto, Marco Gerdol, Giorgio Bertorelle

**Affiliations:** Department of Life Sciences and Biotechnology, University of Ferrara, Ferrara, Italy; Department of Life Sciences, University of Trieste, Trieste, Italy; Department of Life Sciences and Biotechnology, University of Ferrara, Ferrara, Italy; Department of Life Sciences, University of Trieste, Trieste, Italy; Department of Life Sciences and Biotechnology, University of Ferrara, Ferrara, Italy; Department of Life Sciences and Biotechnology, University of Ferrara, Ferrara, Italy; Australian Rivers Institute, Griffith University, Brisbane, Queensland, Australia; Department of Life and Environmental Sciences, Marche Polytechnic University, Ancona, Italy; Research and Innovation Centre, Fondazione Edmund Mach, 38010 San Michele all’Adige, Italy; Center Agriculture Food Environment, University of Trento, 38010 San Michele all'Adige, Italy; Department of Life and Environmental Sciences, Marche Polytechnic University, Ancona, Italy; Australian Rivers Institute, Griffith University, Brisbane, Queensland, Australia; Seqwater, Ipswich, 4305 Queensland, Australia; Seqwater, Ipswich, 4305 Queensland, Australia; Australian Rivers Institute, Griffith University, Brisbane, Queensland, Australia; Department of Biology and Biotechnology, University of Pavia, Pavia, Italy; Department of Life Sciences, University of Trieste, Trieste, Italy; Department of Life Sciences and Biotechnology, University of Ferrara, Ferrara, Italy

**Keywords:** genome size evolution, lungfish, pervasive transcription, relaxation of natural selection, Australian lungfish (*Neoceratodus forsteri*)

## Abstract

Nonadaptive hypotheses on the evolution of eukaryotic genome size predict an expansion when the process of purifying selection becomes weak. Accordingly, species with huge genomes, such as lungfish, are expected to show a genome-wide relaxation signature of selection compared with other organisms. However, few studies have empirically tested this prediction using genomic data in a comparative framework. Here, we show that 1) the newly assembled transcriptome of the Australian lungfish, *Neoceratodus forsteri*, is characterized by an excess of pervasive transcription, or transcriptional leakage, possibly due to suboptimal transcriptional control, and 2) a significant relaxation signature in coding genes in lungfish species compared with other vertebrates. Based on these observations, we propose that the largest known animal genomes evolved in a nearly neutral scenario where genome expansion is less efficiently constrained.

## Introduction

Genome size varies by at least five orders of magnitude in eukaryotic organisms, displaying significant interspecific differences within phyla and even within lower taxonomic rank (e.g., vertebrates; Blommaert 2020). Although this remarkable variation is explained by the different genomic content of noncoding DNA and repeated sequences, among which transposable elements (TEs) usually play a relevant role (Hidalgo et al. 2017; Wright 2017), the evolutionary processes underlying the control (or lack thereof) of genome size in eukaryotes are still debated (Whitney and Garland 2010; Lynch 2011; Lynch and Marinov 2015; Wright 2017).

Adaptive hypotheses assume that the evolution of genome size is the result of natural selection acting on several phenotypic correlates (Cavalier-Smith 1982; Vinogradov 1995). Indeed, although genome size does not correlate with organism complexity (the so-called *C*-value paradox, Thomas 1971; Gregory 2001), it does correlate positively with cell and nucleus size and negatively with cell division rate, which, in turn, may affect the metabolic rate or life cycle complexity (Gregory 2002; Liedtke et al. 2018; Roddy et al. 2021). Alternatively, nonadaptive and (nearly) neutral hypotheses assume that most insertions and deletions are neutral or slightly deleterious, so that they cannot be effectively removed by natural selection when the effective population size (*Ne*) is small, and random drift is consequently large (Lynch and Conery 2003; Wright 2017). Studies explicitly testing the nearly neutral hypothesis led to contrasting results about the role of evolutionary factors, such as *Ne*, in the evolution of genome size (e.g., Mohlhenrich and Mueller 2016; Lefébure et al. 2017; Roddy et al. 2021), leaving the debate still open.

Despite the astonishing diversity of the genome sizes of eukaryotes, species possessing enormous genomes (i.e., >40 Gb) are exceptionally found only in plants (Psilotales in ferns, Liliales, and Santalales in flowering plants) and in lungfish and salamanders among vertebrates (Gregory 2001; Hidalgo et al. 2017). Lungfish genomes and cells are among the largest known in vertebrates (Pedersen 1971), a feature that negatively correlates with their metabolic rates, among the lowest measured for any fish species (Brett 1972; Seifert and Chapman 2006). To trace the rate of expansion of lungfish genome size, Thomson (1972) studied cell size in osteocyte lacunae of fossil osteoclasts, under the assumption that cell size and genome size are correlated (see also Davesne et al. 2021). The fossil cell size trend suggests that genomes were small in the Devonian, and then their size started increasing after the main diversification of the group and the significant decline in taxonomic diversity that occurred in the Carboniferous. Depending on the species, cell size and thus genome size in lungfish reached a plateau between 200 and 100 Ma (Thomson 1972). Recent genomic analyses support this view, indicating that genome size expanded at a rate of 160 Mb/My until ∼200 Ma and more slowly afterwards (Meyer et al. 2021; Wang et al. 2021). The same genomic analysis showed that genomes are large mainly because they accumulated TEs and noncoding DNA. Indeed, repeated sequences account for about 90% of the *Neoceratodus forsteri* genome and at least 61.7% of the *Protopterus annectens* genome. Interestingly, the chromosomes of *N. forsteri*, although considerably expanded, retain the synteny of chordate linkage groups (Meyer et al. 2021).

In this study, we test the hypothesis of (nearly) the neutral evolution of lungfish genome size, whereby a relaxation of natural selection would have allowed the accumulation of noncoding and repetitive genomic elements. Using the newly assembled *N. forsteri* transcriptome in a comparative molecular evolutionary framework, our analyses support a model where a less efficient purifying selection method might have significantly contributed to genomic gigantism in lungfish.

## Results

### Transcriptome Assembly, Annotation, Assessment of Transposon Activity, and Pervasive Transcription

**Fig. 1.**
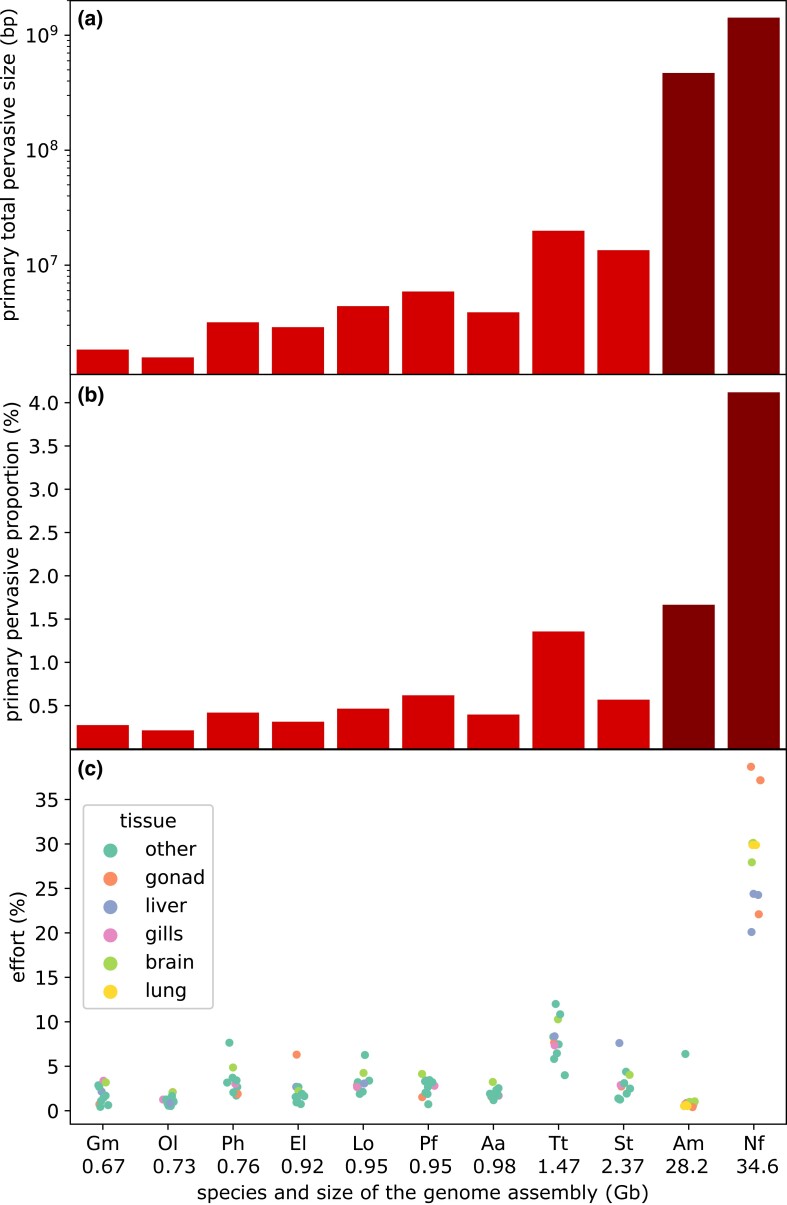
(*a*) The total size of the genomic regions subjected to intergenic pervasive transcription across different species. Please note the logarithmic scale. (*b*) The total fraction of the genome subjected to pervasive transcription, based on detected primary transcripts (i.e., by including introns). (*c*) Total contribution of pervasively transcribed intergenic regions to the global transcriptional effort in different tissues from multiple species. Gonads may refer either to the testis or to the ovary, depending on available data. Gm, *Gadus morhua*; Ol, *Oryzias latipes*; Ph, *Pangasianodon hypophthalmus*; El, *Esox lucius*; Lo, *Lepisosteus oculatus*; Pf, *Perca fluviatilis*; Aa, *Anguilla anguilla*; Tt, *Thymallus thymallus*; St, *Salmo trutta*; Am, *Ambystoma mexicanum*; Nf, *Neoceratodus forsteri*. Species are ordered based on the size of the assembled genome considered in the analyses, from the smallest (left) to the largest (right). Data are available in supplementary tables S1, S5, and S6, Supplementary Material online.

Two adult male specimens of *N. forsteri*, with an estimated age of 45 and 39 years (Fallon et al. 2019), respectively, were sampled in the Brisbane River, southeast Queensland, Australia. We de novo assembled a transcriptome, evaluated its completeness, and performed a functional annotation from the paired end reads obtained from five different tissues of the first individual (Supplementary material online). Assembly metrics (a high fraction of transcripts with full-length coding sequences, 93.76% of vertebrate BUSCOs detected as complete, 5.41% fragmented, and only 0.81% missing) indicated the presence of several protein-coding transcripts that could not be accurately annotated in the reference genome of the same species because of technical constraints (Meyer et al. 2021; supplementary table S3, Supplementary Material online). Although significant TE activity was detected (supplementary table S4 and fig. S1, Supplementary Material online), our analyses also revealed that 189,822 out of 289,539 contigs (65.56% of the total) were categorized as noncoding mRNAs associated with nonrepetitive regions. These results suggest that nearly two-thirds of all assembled expressed sequences in *N. forsteri* may be linked to pervasive noncoding transcription (supplementary fig. S2, Supplementary Material online). A comparative analysis with other bony fish species and with the axolotl, an amphibian with a giant genome, revealed that the total amount of pervasively transcribed genomic sequence was positively correlated with the size of the assembled genomes (Spearman *r_s_* = 0.95, *P* < 10^−4^, fig. 1*a*, supplementary tables S1 and S5, Supplementary Material online). Nevertheless, in the Australian lungfish, such regions spanned a much larger fraction of the genome than in other bony fish (i.e., 8×) or axolotl (2.5×; fig. 1*b*).

These observations were further supported by the contribution provided by pervasive transcription to the total transcriptional effort in multiple tissues. In fact, the proportion of reads mapped to noncoding transcripts unrelated to TE activity, compared with protein-coding transcripts and TEs, was highly prevalent in all lungfish tissues. Little variation was found between the two analyzed individuals, ranging from 24.30% in the liver of the first individual to 38.68% in the testis of the second individual (fig. 1*c*, supplementary table S6, Supplementary Material online). Overall, the testis was the tissue where pervasive transcription was more significant, consistent with the expected relaxed state of chromatin during spermatogenesis, which may increase its accessibility by the RNA polymerase II complex and accessory factors regulating transcription (Villa and Porrua 2023). Widespread intergenic transcription was less prominent in the liver, whereas the lung and brain showed an intermediate level of activity. Based on these data, the Australian lungfish clearly emerged as an outlier compared with all other species, including axolotl, where no more than 6.39% of all transcriptional effort was linked to this class of mRNAs (fig. 1*c*). On average, despite the slightly higher expression observed in *Thymallus thymallus*, mean pervasive transcription accounted for only 3.06% (with a standard deviation of 2.34%) in bony fishes.

### Relaxation of Selection in Lungfish

**Fig. 2.**
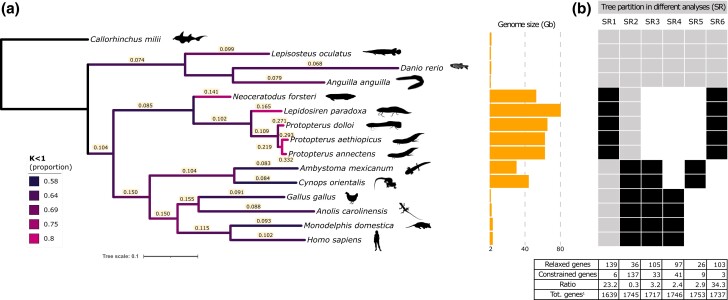
Branch-specific selective pressure (*ω* and *k*) and selection relaxation signature in different tree partitions estimated from 1,790 (or subsets) orthologous genes in 15 vertebrate species, including 5 lungfish. (*a*) Maximum-likelihood phylogeny was inferred using IQ-TREE v1.6.10 (Nguyen et al. 2015) and model selection via ModelFinder (Kalyaanamoorthy et al. 2017; all branches were supported by 100% bootstrap values). The scale bar represents in expected number of nucleotide replacements per site. The numbers shown along each branch are the maximum-likelihood estimates of *ω* obtained with CODEML free-ratio. Branches are colored according to the proportion of potentially relaxed genes (*k* < 1) estimated with RELAX (general descriptive model), increasing from blue to magenta. Orange bars show the genome size for each species (Gb) according to the Animal Genome Size Database (Gregory 2023. http://www.genomesize.com). (*b*) Six different branch partitions (SR1–SR6) defined to infer the number of genes out of the initial 1,790 orthologues showing intensified signals of relaxation (CODEML *ωfg* > *ωbg* ∩ RELAX *k* < 1; “Relaxed genes” in the table) or conservation (CODEML *ωfg* < *ωbg* ∩ RELAX *k* > 1, “Constrained genes” in the table). Black boxes: foreground (CODEML) or test (RELAX) branches, gray boxes: background (CODEML) or reference (RELAX) branches. ^1^Total number of genes that produced an output in both RELAX and CODEML out of 1,790.

We analyzed a set of 1,790 conserved single copy genes (BUSCO) in 15 different species (supplementary table S2, Supplementary Material online), including 5 of the 6 (or 7 according to Carneiro et al. 2021) recognized lungfish species (fig. 2*a*), while for *Protopterus amphibious*, genomic data are not available. We followed two approaches to detect positive and relaxed selection (CODEML, Yang 1998 and RELAX, Wertheim et al. 2015). Only those genes that showed a significant and consistent signature in both approaches were considered for further analyses.

The intersection of CODEML and RELAX results showed that 139 and 6 lungfish genes were more relaxed and more conserved, respectively, compared with the rest of the tree (4 fish and 6 tetrapods, SR1; fig. 2*b*, supplementary fig. S3, Supplementary Material online).

The high number of relaxed genes is surprising, considering that BUSCO genes are evolutionarily conserved. Alternative partitions (SR2-6, fig. 2*b*) were tested to confirm that the lungfish clade shows evidence of selective relaxation that other groups do not show. A comparison of the proportion of significantly more relaxed and significantly more constrained genes in the different partitions showed that the purifying selection process appeared to be significantly weaker (*χ*^2^ test *P* < 0.001) in the lungfish clade. Furthermore, single-branch analyses within the lungfish group showed the same trend and revealed a linage-specific rather than gene-specific relaxation signal, as expected for a random, nondirectional effect (supplementary table S7 and fig. S4, Supplementary Material online). In contrast, other species with giant genomes, the urodeles *Ambystoma mexicanum* and *Cynops orientalis*, behave similarly to other tetrapods (fig. 2*b*). When single branches were considered in the RELAX analysis (general descriptive model), lungfish showed a higher proportion of loci with *k* < 1 (i.e., relaxation of selection) than the rest of the tree (fig. 2*a*). Finally, branch-specific *ω* values obtained on the concatenated 1,790 genes (free-ratio model, CODEML, fig. 2*a*) further supported a significant trend toward higher *ω* values (indicative of relaxation of selection) in lungfish (Mann–Whitney *U* test, *P* < 0.01).

Under strong relaxation conditions, even generally conserved genes are expected to accumulate deleterious mutations, possibly leading to amino acid replacements at key protein sites. To test whether this was the case in the putatively relaxed lungfish lineages, we identified and counted, in each of the 15 terminal branches of the tree in figure 2*a*, the private amino acidic substitutions for each of the 15 lineages. These changes were then classified as either radical or conservative according to the conservative-radical index (Sharbrough et al. 2018; supplementary fig. S5, Supplementary Material online). With the exception of *P. annectens*, which is also a lungfish species with a larger *ω*, lungfish species showed trends that are similar to the rest of the tree, suggesting that the core gene set analyzed, although relaxed, is not drifting toward pseudogenization.

### Biological Functions in Lungfish Relaxed and Constrained Genes

The set of relaxed genes was not associated with a statistically significant enrichment of specific biological functions, with the sole exception of the ten GO terms listed in supplementary table S8, Supplementary Material online, which mostly related to protein turnover. Among the six genes more constrained in lungfish, *SMC5* (structural maintenance of chromosomes 5) is particularly interesting, given that its protein product is involved in the repair of DNA double-strand breaks by homologous recombination, telomere maintenance, and sister chromatid cohesion during prometaphase and mitotic progression, functions that could be crucial in the presence of giant genomes.

## Discussion

The transcriptome of *N. forsteri* is characterized by a strong background noise caused by the abundance of short, pervasively transcribed RNAs, the expression of which is not associated with coding genes, or caused by TE activity. Pervasive transcription is a common feature of eukaryotic genomes, occasionally providing the raw material for new genes and long noncoding RNAs (Oss and Carvunis 2019; Palazzo and Koonin 2020) but generally representing a by-product of transcription events whose excess is contained through a specific cellular “toolbox” (Jensen et al. 2013; Koonin 2016). In this framework, pervasive transcription is expected to increase when selection is less efficient in controlling this costly, inefficient, and possibly harmful molecular mechanism. The relevant pervasive transcription observed in lungfish tissues, together with the relaxation signal shown by conserved coding genes analyzed by different comparative approaches, suggests that lungfish genomes have been under reduced selective constraints compared with those of other vertebrate species.

Although multiple lines of evidence support this interpretation, we describe some limitations of our analysis and explain why we believe that their impact on the general conclusion is negligible.

First, the large evolutionary distance between lungfish species and between lungfish and other vertebrates, and the huge size of lungfish genomes, may have produced genome annotation errors. In particular, our pipeline could not discriminate between long intergenic noncoding RNAs (lincRNAs), which are still scarcely known and rarely annotated in nonmodel organisms, and proper transcriptional leakage, intended as a phenomenon linked to a scarce control of the transcription molecular machinery (Villa and Porrua 2023). However, it is reasonable to assume that a similar number of lincRNA genes (close to zero) has been properly annotated in the genomes of all the species taken into account, and therefore, it is unlikely that it could skew our estimates. Second, when looking for a signature of relaxed selection in lungfish using the ratio d*N*/d*S*, the deep divergence times (e.g., *N. forsteri*/other lungfish about 230 My, lungfish/tetrapods: about 400 My, Kumar et al. 2022) may have introduced bias because of the saturation at third codon positions and the consequent underestimation of the synonymous substitution rate. The extent to which saturation affects the robustness of codon-based models is controversial (Seo and Kishino 2008; Gharib and Robinson-Rechavi 2013; Weber et al. 2014), but high-sequence divergence alone does not appear to be a serious problem if the alignment is reliable (Yang and dos Reis 2011). The strength of the relaxation signal in lungfish is reinforced by the conservative nature of branch models toward the selective constraint hypothesis. In fact, d*N*/d*S* was estimated by averaging nucleotide substitution rates over sites in the protein (Yang and dos Reis 2011) and by including in the data set only conserved gene sequences that were further trimmed by removing poorly aligned regions. Finally, we ruled out that the lungfish signal was random by analyzing different partitions of the tree (fig. 2*b*, supplementary methods, Supplementary Material online), and we have been extremely conservative in describing as *relaxed* only those genes detected by both approaches, CODEML and RELAX.

In conclusion, our results on pervasive transcription and the accumulation of nonsynonymous variants are robust and consistent with the prediction from the nearly neutral theory of the inverse relationship between the strength of purifying selection and genome size (Lynch and Conery 2003), at least when fish species are compared. As previously observed (Mohlhenrich and Mueller 2016), the two amphibians with giant genomes included in our study, *A. mexicanum* and *C. orientalis*, did not show the same signals, thus arguing against a simple and universal correlation. Consistent with a *nearly* neutral scenario, in which purifying selection is still acting on variants with a large selection coefficient, the genes analyzed were not enriched for radical amino acid changes in the five lungfish species.

Why do lungfish show reduced purifying selection that possibly allowed genome expansion? A reasonable potential explanation is a small, long-term population size. The decline of the lungfish group started in the Carboniferous (Jorgensen and Joss 2016) when, according to fossil bones, genome size started to increase (Thomson 1972) and the number of species started to decrease. The reasons for such a decline in species diversity are not known, but if this process also reduced the population sizes of the surviving species, enhanced drift effects and reduced purifying selection may have allowed genome size expansion through reduced control of TE proliferation (Lockton et al. 2008; Oggenfuss et al. 2021). Genome expansion may trigger evolutionary radiation and/or the evolution of new gene functions (Davesne et al. 2021), but the negative effects of TE proliferation and genomic expansion (Boissinot et al. 2006; Hollister and Gaut 2009; Wei et al. 2022) probably prevailed in lungfish history. Our data suggest a causal link between reduced population size and increased genome size through relaxed selection and TE proliferations.

## Materials and Methods

A detailed account of the methods can be found in the supplementary text, Supplementary material online.

## Supplementary Material

msad193_Supplementary_Data

## Data Availability

The data presented in this study are openly available in NCBI BioProject; Accession: PRJNA605733. A novel custom bioinformatic script used in this work to identify and count private nonsynonymous changes in phylogeny is available at https://github.com/rsbiello/Lungfish_relax.
