# Supplementary material for: Relaxation of Natural Selection in the Evolution of the Giant Lungfish Genomes
Source: Mol Biol Evol. 2023 Sep 6;40(9):msad193. doi: 10.1093/molbev/msad193 (PMC10503785; doi:10.1093/molbev/msad193)
Supplement: msad193_Supplementary_Data [file msad193_supplementary_data.pdf]

Relaxation of natural selection in the evolution of the giant lungfish genomes

Fuselli S[1], Greco S[2], Biello R[1], Palmitessa S[2], Lago M[1], Meneghetti C[1], McDougall C[3], Trucchi E[4], Rota Stabelli O[5,6], Biscotti MA[4], Schmidt DJ[3], Roberts DT[7], Espinoza T[7], Hughes JM[3], Ometto L[8], Gerdol M[2] and Bertorelle G[1]

[1]Department of Life Sciences and Biotechnology, University of Ferrara, Ferrara, Italy

[2]Department of Life Sciences, University of Trieste, Trieste, Italy

[3]Australian Rivers Institute, Griffith University, Brisbane, Queensland, Australia

[4]Department of Life and Environmental Sciences, Marche Polytechnic University, Ancona, Italy

[5]Research and Innovation Centre, Fondazione Edmund Mach, 38010 San Michele all'Adige, Italy

[6]Center Agriculture Food Environment, University of Trento, 38010 San Michele all'Adige, Italy

[7]Seqwater, Ipswich, 4305 Queensland, Australia

[8]Department of Biology and Biotechnology, University of Pavia, Pavia, Italy

Corresponding email address: fss@unife.it, ggb@unife.it

Supplementary material online

Table of contents

1. Supplementary methods

1.1. Collection of samples, library preparation and transcriptomic sequencing

1.2. *De novo* transcriptome assembly and functional annotation

1.3. Assessment of transposon activity

1.4. Assessment of pervasive transcription

1.5. Orthologous sequence recovery

1.6. Inference of phylogenetic tree

1.7. Molecular evolution analyses: dN/dS estimates

1.8. Private NS changes

1.9. GO enrichment of genes under different evolutionary rates

2. Supplementary results

2.1 Sequencing, *de novo* transcriptome assembly and identification of non-coding transcripts

2.1.1. Evaluation of transposable element activity

2.1.2. Impact of pervasive transcription on global transcriptional activity

2.2 Relaxation of selection in lungfish and private nonsynonymous changes

2.3 GO enrichment of genes under different evolutionary rates.

3. Supplementary references

1. Supplementary Methods

1.1 Collection of samples, library preparation and transcriptomic sequencing

Two adult male specimens of *N. forsteri*, with estimated age of 45 and 39 years (Fallon et al. 2019), respectively, were sampled with an electrofishing boat in the Brisbane River, southeast Queensland, Australia. The specimens were anaesthetized with AQUI-S anesthetic, transported on ice back to the laboratories of the Griffith University. The required permits for sampling for scientific purposes were obtained from the Queensland authorities (Australian Ethics Committee protocol number ENV/03/18/AEC; Queensland fisheries permit number 182081).

The specimens were dissected and samples from all the main organs and tissues were immediately placed in RNAlater solution (Sigma) and stored at -80°C until extraction. Homogenized lung, brain, liver and testis samples were selected for total RNA extraction. RNA was extracted using Tri Reagent (Sigma) following the manufacturer's protocol, using 1-Bromo-3-chloropropane for phase separation and 50:50 isopropanol and high salt solution (0.8M sodium citrate, 1.2M sodium chloride) for precipitation. RNA integrity was assessed on an Agilent 4200 Tapestation High Sensitivity ScreenTape. To provide a broader representation of the landscape of mRNAs expressed in lungfish, the RNAs extracted from 5 additional tissues (i.e. kidney, blood, eye, muscle, and heart) in the first individual were mixed in equimolar quantities to obtain a pooled sample.

Upon an initial check that a suitable quality and quantity of extracted RNAs was available for all samples, carried out with Nanodrop 2000 (Thermo Fisher Scientific, Waltham, MA, USA) and Tapestation 2200 (Santa Clara, CA, USA) instruments, barcoded stranded polyA-selected cDNA libraries compatible with Illumina sequencing were prepared with a TruSeq Stranded mRNA kit (Illumina, San Diego, CA, USA).

All libraries were prepared and sequenced by Omega Bioservices (Norcross, GA, USA) and Australian Genome Research Facility (AGRF, Melbourne, VIC, Australia). The libraries obtained from the first individual were sequenced on a HiSeq 2500 platform (Illumina, San Diego, CA, USA) with a 2x150 paired-end (PE) strategy, providing the reference material for the subsequent *de novo* transcriptome assembly process. The libraries obtained from the second individual were sequenced on a NovaSeq 6000 platform (Illumina, San Diego, CA, USA) using a 1x100 strategy, generating single-end (SE) reads that provided a biological replicate for an accurate evaluation of transcriptional activity.

1.2 *De novo* transcriptome assembly and functional annotation

All raw reads were demultiplexed, quality-checked, and trimmed according to base-calling quality scores and the presence of residual Illumina sequencing adaptors and barcodes using FastQC v. 0.11.9 (Andrews 2010). The first 15 nucleotides at the 5'end of each read were trimmed to remove the compositional bias observed in this region due to the non-random adapter ligation (Wright et al. 2019). Trimmed reads shorter than 75 bp were discarded.

The trimmed PE reads from the first individual were *de novo* assembled using the Oyster River Protocol v.2.3.1 (MacManes 2018), which combines the outputs of three different assemblers, i.e. Trinity (Grabherr et al. 2011), SPAdes (Bankevich et al. 2012) and TransABySS (Robertson et al. 2010) to generate a non-redundant highly representative transcriptome. Poorly expressed transcripts, possibly resulting from exogenous contamination or otherwise not biologically relevant, were removed by setting the TPM_FILT parameter to 1.

Contigs matching ribosomal RNA or encoded by mitochondrial DNA were detected with BLASTn (Altschul et al. 1990), based on an e-value threshold equal to $1E^{-10}$, and removed. The nucleotide sequences with GenBank accession ID AJ584642.1 (complete mitochondrial genome) (Arnason et al. 2004), U34338.1 (28S rRNA) and KJ784395 (18S rRNA) (Zardoya and Meyer 1996) were used as queries for this task.

The completeness of the transcriptome assembly was evaluated by the analysis of a highly conserved set of single-copy orthologous genes shared by all vertebrates, extracted from OrthoDB v.10 (Kriventseva et al. 2008), using BUSCO v.3 (Simão et al. 2015), computing the fraction of complete, fragmented and missing orthologs.

Functional annotation was performed with annotaM (https://github.com/54mu/annotaM), which assigned Gene Ontology terms (Ashburner et al. 2000) to each contig based on the retrieval of BLASTx hits against the UniProtKB/Swiss-Prot database (e-value threshold = 1×10^{-5}), and Pfam conserved domains (Punta et al. 2012) with HMMer v.3.1b2 (Finn et al. 2011) (using default settings).

1.3 Assessment of transposon activity

A *de novo* library of the repeated elements found in the reference genome of *N. forsteri* (Meyer et al. 2021) was generated with RepeatScout v.1.0.5 (Price et al. 2005). The output of this process was analysed with RepeatMaskerv.4.1.2 (Smit 2015), which allowed the removal of the sequences observed less than 10 times in the entire genome, and therefore unlikely to represent *bona fide* transposable elements. All the elements included in the filtered library were then classified with the Transposon Classifier RFSB tool included in the TransposonUltimate bundle (Riehl et al. 2022).

The trimmed reads obtained from lung, brain, liver, and testis in both individuals were individually mapped against the *de novo* transcriptome assembly and the filtered repeat library with Salmon v.1.3.0 (Patro et al. 2017). The global transcriptional activity of transposable elements was computed by calculating the fraction of the reads mapped to the repeat library for all samples, relative to the number of reads mapped to the complete transcriptome assembly.

1.4 Assessment of pervasive transcription

Different approaches were used to evaluate the rate of pervasive transcription in lungfish. First, the contigs included in the newly generated *de novo* transcriptome assembly were matched with the complete gene

sequences annotated in the reference genome (Meyer et al. 2021) to identify non-coding transcripts putatively derived from intergenic regions. This task was carried out with a BLASTn search, based on an e-value threshold of 1×10^{-10} and included all annotated exon sequences. Assembled contigs lacking significant matches were further inspected for the presence of Open Reading Frames (ORFs) larger than 100 codons with Transdecoder v.5.5.0, to remove genuine protein-coding transcripts encoded by unannotated or wrongly annotated genes, based on the previous report of the suboptimal BUSCO score of the reference genome annotation (Meyer et al. 2021). The remaining contigs were also screened with RepeatMaskerv.4.1.2, removing those characterized by the presence of any element included in the repeat library described in the previous section, ruling out an association with transposable element activity. Finally, the presence of transcripts completely embedded within the introns of annotated genes was assessed with a BLASTn search, using the same e-value threshold listed above, only retaining hits with 100% query coverage. All resulting transcripts lacked protein-coding potential, significant sequence homology with the annotated gene set and repeated elements, being therefore considered as the likely product of pervasive transcription.

To further investigate the impact of this phenomenon at a comparative level, gene annotations and multiple publicly available RNA-Seq datasets were retrieved from NCBI for a number of representative bony fish species and for one amphibian with a giant genome, the axolotl *Ambystoma mexicanum* (see table S1 for the complete list of SRA accession IDs). To improve the detection of pervasively transcribed genomic regions in *N. forsteri*, the sequencing data generated in this study was complemented with additional publicly available libraries generated from a juvenile individual of undetermined sex, which only included the brain, liver and gonad tissues (BioProject accession ID: PRJNA645042).

A snakemake (Köster and Rahmann 2018) pipeline was built by combining several tools, i.e., fastp (Chen et al. 2018) for raw read trimming, STAR (Dobin et al. 2013) for mapping trimmed reads against the reference sequence datasets, samtools (Danecek et al. 2021), bed tools (Quinlan and Hall 2010) and bedops (Neph et al. 2012) for handling and analysing mapping files. Briefly, mapping only retained reads marked with a single non-ambiguous alignment against the reference genome (i.e. those flagged with NH:i:1), and all multi-mapping read were discarded. To prevent the inclusion of misalignments, stringent mapping parameters were used to exclude low scoring matches (-q parameter was set to 20), and only reads mapped in pairs were used to assess pervasive transcription. The read mapping files derived from the alignment between all available RNA-seq datasets and annotated reference genomes for each species were analysed with the aim to identify stretches of intergenic genomic sequence displaying high mapping coverage (i.e. at least 20X higher than expectations in case of random mapping), indicative of transcriptional activity. To further mitigate the impact of non-specific mappings, only regions longer than 254 nucleotides (i.e. the median exon length of the genes annotated in *N. forsteri*) were taken into account. The identified regions were then used to compute the fraction of the genome subjected to pervasive

transcription, both in terms of primary and spliced transcript size (i.e. by including or excluding introns, respectively).

Table S1. Raw read accession IDs used in the pervasive transcription comparative analysis.

**Species**	**SRA accession of raw reads**
Gadus morhua	SRR2045415, SRR2045416, SRR2045417, SRR2045418, SRR2045419, SRR2045420, SRR2045421, SRR2045422, SRR2045423, SRR2045424, SRR2045425
Esox lucius	SRR1533651, SRR1533652, SRR1533653, SRR1533654, SRR1533655, SRR1533656, SRR1533657, SRR1533658, SRR1533659, SRR1533660, SRR1533661
Perca fluviatilis	SRR1533685, SRR1533686 , SRR1533687, SRR1533688, SRR1533689, SRR1533690, SRR1533691, SRR1533692, SRR1533693, SRR1533694, SRR1533695
Lepisosteus oculatus	SRR1524250, SRR1524251, SRR1524252, SRR1524253, SRR1524254, SRR1524255, SRR1524256, SRR1524257, SRR1524258, SRR1524259, SRR1524260
Ambystoma mexicanum	SRR15610218, SRR15610219, SRR15610228, SRR15610229, SRR15610238, SRR15610239, SRR15610240, SRR15610241, SRR15610252, SRR15610265, SRR15610266, SRR15610267, SRR15610268, SRR7396731
Oryzas latipes	SRR1524271, SRR1524272, SRR1524273, SRR1524274, SRR1524275, SRR1524276, SRR1524277, SRR1524278, SRR1524279, SRR1524280, SRR1524281
Thymallus thymallus	SRR1533662, SRR1533663, SRR1533664, SRR1533665, SRR1533666, SRR1533667, SRR1533668, SRR1533669, SRR1533670, SRR1533671, SRR1533672
Pangasianodon hypophtalmus	SRR1533640, SRR1533641, SRR1533642, SRR1533643, SRR1533644, SRR1533645, SRR1533646, SRR1533647, SRR1533648, SRR1533649, SRR1533650
Salmo trutta	SRR1532777, SRR1532778, SRR1532779, SRR1532780, SRR1532781, SRR1532782, SRR1532783, SRR1532784, SRR1532785, SRR1532786, SRR1532787
Anguilla anguilla	SRR1532756, SRR1532757, SRR1532758, SRR1532759, SRR1532760, SRR1532761, SRR1532762, SRR1532763, SRR1532764, SRR1532765, SRR1532766

1.5 Orthologous sequence recovery

To estimate the rate of evolution of the lungfish lineage, we analyzed the coding sequence (CDS) from a set of orthologous genes expected to be highly conserved and not subjected to duplication in all vertebrates. These sequences were extracted from the genomes (where available) and/or transcriptomes of several selected species (table S2). Transcriptome assemblies were retrieved from the NCBI TSA database or, alternatively, *de novo* assembled as described above for the Australian lungfish, using the raw RNA-seq data retrieved from the SRA database as an input. Orthologous genes were recovered with BUSCO v.3 (Simão et al. 2015) based on the matches obtained against the vertebrate OthoDB v.10 dataset (Kriventseva et al. 2008). Only unambiguous matches (i.e. complete and not duplicated) were subjected to further analysis. The extracted CDS for each ortholog were aligned with MACSE v.0.9b1 (Ranwez et al. 2011) and alignments were manually inspected to identify extremely divergent sequences in one or a few species, which may indicate the presence of a paralogous gene or of incorrect gene predictions in that (or those) specific lineages. In those cases, the entire gene was removed from the dataset. Finally, the multiple sequence alignments were concatenated and trimmed with BMGE (Criscuolo and Gribaldo 2010), run in a codon mode (selecting a BLOSUM95 substitution matrix), removing all gapped positions.

Table S2. Species names and respective transcriptome resources for orthologous sequence recovery and dN/dS analyses.

species	Reference or accession number
Ambystoma mexicanum	Smith et al. 2019
Anguilla anguilla	Vertebrate Genome Project (Rhie et al. 2021)
Anolis carolinensis	Alföldi et al. 2011
Callorhinchus milii	Venkatesh et al. 2014
Cynops orientalis	Biscotti et al. 2020
Danio rerio	Howe et al. 2013
Gallus gallus	International Chicken Genome Sequencing Consortium (Hillier et al. 2004)
Homo sapiens	NCBI genome assembly GRCh38.p13
Lepidosiren paradoxa	Nogueira et al. 2016
Lepisosteus oculatus	Braasch et al. 2016
Mondelphis domestica	Mikkelsen et al. 2007
Neoceratodus forsteri	*De novo* assembled (this work; NCBI BioProject PRJNA605733)
Protopterus aethiopicus	NCBI BioProject PRJNA473927
Protopterus annectens	Biscotti et al. 2016
Protopterus dolloi	Takezaki and Nishihara 2017

1.6 Inference of phylogenetic tree

A maximum-likelihood tree was inferred with IQ-TREE v1.6.10 ((Nguyen et al. 2015) using a set of 1790 conserved single-copy genes (BUSCO) from 15 vertebrate species. The dataset was partitioned by a single gene and a substitution model was selected for each partition by the built-in ModelFinder option MFP+MERGE (Kalyaanamoorthy et al. 2017) and 1,000 ultrafast bootstrap replicates (Hoang et al. 2018) were performed. Final figures were made using iTOL (Letunic and Bork 2019).

1.7 Molecular evolution analyses: dN/dS estimates

Starting from the dataset of 1,790 BUSCO genes, dN/dS rates (ω) were computed with CODEML, included in the PAML v.4.8 package (Yang 2007), testing different models. In particular, to identify signatures or relaxation of natural selection we performed a branch test, which can detect a different rate of evolution in only one (or a group of) lineage of a tree (Yang 1998). In this analysis, the branches of the phylogenetic tree are divided into foreground and background branches and selection is tested with a comparison of two models. We used the contrast of the one-ratio model in which the foreground and the background have the same ω value ($\omega_{fg} = \omega_{bg}$, the null) and the two-ratio model in which the foreground and the background have two different ω values ($\omega_{fg} \neq \omega_{bg}$, the alternative). In particular, $\omega_{fg} > \omega_{bg}$ indicates positive or relaxed selection acting on the foreground branches, while $\omega_{fg} < \omega_{bg}$ indicates an excess of constraint in the foreground. In the first analysis, lungfish (foreground) were compared with the rest of the tree (background, SR1 in fig. 2b main text). Additional partitions of the tree (SR2-SR6) were also considered to confirm that the signal showed by the lungfish clade was robust. Each of the new partitions was designed to account for a specific bias or clade-specific effect. In detail, to confirm that the result of SR1 was lungfish-specific, tetrapods and lungfish were switched, and tetrapods were compared with the rest of the tree (SR2). To further control for the possible effect of having lungfish in the background, tetrapods were compared with fish, lungfish excluded (SR3). The next two partitions, SR4 and SR5, were designed to evaluate the evolutionary signal of the two giant genome urodeles (*A. mexicanum* and *C. orientalis*). Finally, lungfish were compared with the other fish of the dataset for direct comparison with the results of SR3-SR5 analyses. We also used each lungfish branch as foreground branch against the rest of the phylogeny in 9 independent analyses. To identify whether the relaxation signature was gene specific (i.e. the same genes are relaxed in all the lungfish branches) or branch specific, we tested if the overlap between the candidate genes identified in the species-specific foreground branch and in the entire lungfish clade was significantly larger than expected. Significant results are expected when the signal at the clade level is due to a lineage-specific relaxation.

The statistical significance of the differences in ω observed in different tree partitions was evaluated with a likelihood-ratio test (LTR). The resulting *p-value* was corrected for multiple testing with the Benjamini-

Hochberg method (False Discovery Rate, FDR, Benjamini and Hochberg 1995) through the p.adjust function of R v3.6.2 (R Core Team 2019) with a significance threshold of FDR < 0.01. Finally, we calculated branch-specific ω values after concatenating the 1,790 genes, using the free-ratio model implemented in CODEML (Yang 2007).

The same dataset consisting of 1,790 genes and 15 species, as well as the same tree partition scheme (fig. 2b, main text), were considered in a second set of analyses more specifically designed for inferring relaxation of natural selection. These analyses were performed with the software RELAX implemented in the HyPhy v2.5.27 package (Wertheim et al. 2015). The method is based on BS-REL (Branch-Site Random Effects Likelihood) models, developed to improve previous branch-site models, which often generated false positives and false negatives when assumptions were violated (Kosakovsky Pond et al. 2011; Wertheim et al. 2015). The approach is based on the principle that relaxation pushes all ω categories toward neutrality (ω = 1) reducing the intensity of both positive (ω>1) and purifying (ω<1) natural selection. Similar to CODEML analysis, a priori partitions in the phylogeny are defined with a test group, consisting of the branches of interest (foreground in CODEML), compared with the reference group (background in CODEML). The method estimates a discrete distribution of ω shared by all branches of the phylogeny, defined by three classes of ω (ω1, ω2, ω3) to each of which it assigns a certain proportion of sites in the sequence (p1, p2, p3). The test constrains the distribution in the test and reference branches so that $\omega_T = \omega_R^k$, and each class of ω in the test partition is obtained by raising the corresponding class in the reference partition to the power of k. k defines how the intensity of selection in the test branches (T) varies with respect to the reference branches (R) and always takes values greater than or equal to 0. An LRT test is then performed between a null model in which k = 1, i.e., $\omega_T = \omega_R$, and an alternative model in which k is a free parameter. In this work, the resulting *p-value* was corrected for multiple testing with the Benjamini-Hochberg method and a significance threshold of FDR < 0.01 (False Discovery Rate, FDR, Benjamini and Hochberg 1995). Similar to the CODEML free-ratio, RELAX also provides a General Descriptive Model that infers how the relative intensity of selection varies along the phylogeny, without defining a priori partitions within the tree. In this model, all branches share a single distribution of ω, but each branch has its own k parameter that intensifies (k > 1) or relaxes (k < 1) that distribution and can take values between 0 and 50 (Wertheim et al. 2015). We performed this analysis for the 1,790 genes, obtaining a branch-specific k parameter for each gene. We then calculated the proportion of putatively relaxed genes, i.e. genes with k<1 relative to the total number of genes that produced a RELAX result (fig. 2a, main text).

1.8 Private NS changes

We translated the aligned coding sequences used for CODEML and RELAX analyses into their respective amino acid sequences, and we identified in each of the 15 terminal branches of the tree in fig. 2a the positions where 14 lineages share the same amino acid, while a single lineage carries an alternative residue

(script available at https://gitlab.com/54mu/enrichment_test). These private amino acid changes were then classified into either radical or conservative according to the Conservative-Radical Index (CRI, Sharbrough et al. 2018). Briefly, seven different amino acid classification schemes are used to evaluate rates and patterns of radical and conservative amino acid evolution. Each scheme highlights different amino acid properties that are likely to shape protein evolution, for example, amino acid charge, three-dimensional structure, changes between polar and nonpolar amino acids, volume and aromaticity. The overall index of the degree of amino acid change CRI is calculated for each possible amino acid change by averaging across the seven amino acid classification schemes (radical changes assigned a value = 1.0, conservative changes assigned a value = 0.0).

1.9 GO enrichment of genes under different evolutionary rates.

Gene Ontology terms relative to each BUSCO id were retrieved from OrthoDB v.10 (Kriventseva et al. 2019). The enrichment tests were performed with a hypergeometric test, run on the relaxed and constrained gene sets, implemented as a python script (available at https://gitlab.com/54mu/enrichment_test) using an FDR-corrected *p-value* threshold < 0.05 and Observed-Expected threshold > 2 for the selection of significant results.

2. Supplementary Results

2.1 Sequencing, *de novo* transcriptome assembly and identification of non-coding transcripts

The sequencing of the five libraries from the first lungfish individual yielded a total of 197.6M raw PE reads, which were roughly reduced by 3.5% following the trimming procedure, leading to a total of 190.8M clean reads. These were subdivided among the five libraries as follows: 35.9M reads for brain, 43M reads for liver, 37.8M reads for lung, 39M reads for testis and 35.1M reads for the mixed library (table S3). The four libraries obtained from the second lungfish individual generated a total of 161.6M raw SE reads, reduced by 2.2% to 158.1M by the trimming process, for a final set of 43.2M reads for brain, 31.5M reads for liver, 45.5M reads for lung and 37.9M reads for testis.

The *de novo* assembly produced a total of 289,539 contigs, with a length range comprised between 200 and 18,641 nucleotides (mean and median length = 516.33 and 290 nt, respectively, N50 = 778 nt, with 2,522 contigs longer than 5 Kb, see table S3). In spite of a high proportion of short assembled contigs, the transcriptome assembly was found to include a high fraction of transcripts with full-length CDSs and to be highly representative of the complete landscape of protein-coding mRNAs expected to be expressed in this species across a broad range of tissues and physiological conditions. Overall, 917 out of the 978 vertebrate BUSCOs (93.76%) were detected as complete, 53 (5.41%) were fragmented and just 8 (0.81%) were missing. Complete/fragmented and complete/missing ratios were 17.30 and 114.63, respectively.

In further support of the high quality of the *de novo* assembled transcriptome, high mapping rates were observed for nearly all libraries. These were 85.44% (brain), 89.70% (brain), 92.51% (lung) and 90.07% (testis) in the first individual and 82.14% (brain), 92.84% (liver), 91.61% (lung) and 92.14% (testis) in the second individual. The only exception was the library obtained from mixed tissues in the first individual, where the mapping rate was 61.63%, possibly owing to a lower quality of extracted RNA.

The functional annotation associated 24,318 contigs (8.40% of the total) with Gene Ontology terms and 14,329 contigs (4.95% of the total) with Pfam conserved domains. In summary, 24,473 contigs (8.45% of the total) could be associated with a functional annotation (table S3).

Owing to this particularly low annotation rate and the high number of *de novo* assembled transcripts, our assessment of protein-coding potential, overlap with annotated genes in the reference *N. forsteri* genome and presence of repeated elements led to the identification of a total of 189,822 contigs (65.56% of the total) denoting non-coding mRNAs unrelated with TE activity. Out of these, only 1,528 (0.53% of the total) could be tentatively associated with intronic polyadenylated transcripts, even though the present availability of Illumina libraries only and the lack of a detailed map of polyadenylation sites prevented to discriminate such transcripts from fragments of aberrant mRNAs retaining introns.

These results suggest that nearly two-thirds of all assembled expressed sequences in *N. forsteri* (accounting for 34.78% of the complete transcriptome assembly size) may be linked with pervasive non-coding transcription.

Table S3. Summary of the sequencing and *de novo* transcriptome assembly metrics.

sequencing statistics			
library	raw reads (M)	trimmed reads (M)	mapping rate (%)
brain1	37	35.9	85.4%
brain2	44.7	43.2	82.1%
liver1	44.3	43	89.7%
liver2	32.4	31.5	92.8%
lung1	39.2	37.8	92.5%
lung2	46.5	45.5	91.6%
testis1	40.6	39	90.1%
testis2	38	37.9	92.1%
mix	36.5	35.1	61.6%
de novo assembly statistics			
number of contigs	289,539		
mean length	516.3		
median length	290		
N50	778		
contigs longer than 5Kb	2522		
contigs associated with GO terms	24,318 (8.4%)		
contigs associated with Pfam domains	14,329 (4.95%)		
annotated contigs	24,473 (8.45%)		
complete BUSCOs	93.76%		
fragmented BUSCOs	5.41%		
missing BUSCOs	0.81%		
complete/fragmented BUSCO rate	17.30		
complete/missing BUSCO rate	114.63		
contigs linked with pervasive transcription	189,822 (65.56%)		

2.1.1 Evaluation of transposable element activity

Although the strategy used for the identification of pervasively transcribed mRNA described above had already excluded an association with TE activity, we performed an additional analysis to further support the lack of involvement of these elements in the widespread transcription of intergenic regions. Based on the newly generated repeat library (table S4), we computed the global contribution of different classes of transposons to the transcriptional activity observed in brain, liver, lung, and testis in the two analyzed lungfish individuals. Albeit this analysis evidenced a significant variation both among different classes of transposons and between the two biological replicates (with the first individual showing a higher TE activity), TEs cumulatively only accounted for a minor fraction of global transcription in all samples, averaging 1.02% of the total. In general, the brain was the tissue characterized by the highest transposon

activity, reaching 2.08% in individual 1, whereas in the majority of the other samples these values were lower than 1%, with the minimum (0.49%) recorded in the testis from individual 2 (fig. S1).

Table S4. number of sequences in library for each class of transposable elements.

			N# elements in library
superClass	Class	subClass	
DNATransposon	DNATransposon	Helitron	109
		MITE	1,067
	TIR	CMC	846
		Novosib	26
		Sola	806
		Tc1-Mariner	917
		Zator	8,682
		hAT	1,751
Retrotransposon	LTR	Copia	1,169
		ERV	585
		Gypsy	2,606
	Non-LTR	LINE	135
		SINE	32

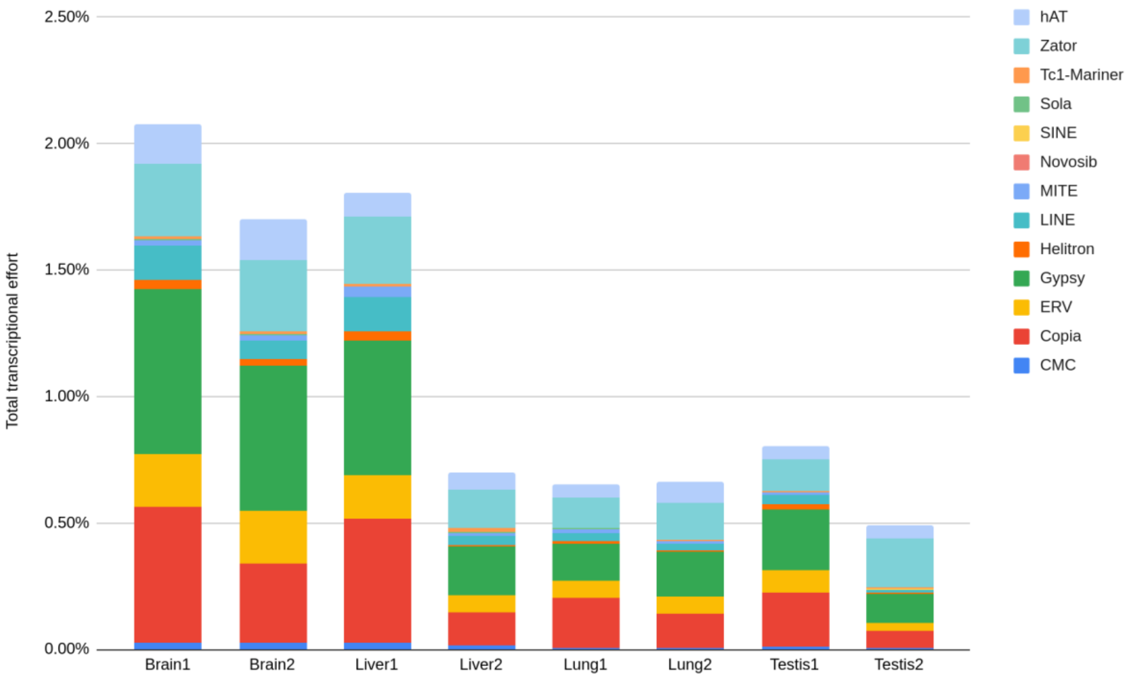

Figure S1. Total transcriptional effort ascribable to transposable elements, displayed as the fraction of reads mapped against the repeat library, with a breakdown of the relative contribution of the main TE subclasses (see table S4 for further details).

2.1.2 Impact of pervasive transcription on global transcriptional activity

In lungfish, we could detect that approximately 1.42 Gb of intergenic genomic sequence was subjected to pervasive transcription, accounting for 4.12% of the total genome assembly size, which is consistent with the identification of a very high number of contigs presumably derived from pervasive transcription in the *de novo* assembled transcriptome (fig. S2). This estimate takes into account the full size of the inferred primary transcripts (i.e. by including introns in the computation), whereas both the spliced size (44.53 Mb) and genomic coverage (0.13%) were significantly smaller, as expected from the very large reported intron size observed in *N. forsteri* (Meyer et al. 2021). The comparative analysis carried out by analyzing the same phenomenon in multiple other bony fish species, as well as in axolotl, an amphibian similarly characterized by a giant genome revealed, as expected, a tendency of larger genomes to display a proportionally larger total amount of pervasively transcribed genomic regions (fig. 1a, main text, table S5), (Spearman rs =0.95, P < 10-4). Nevertheless, the fraction of the genome subjected to pervasive transcription was quite uniform across all bony fish species, regardless of genome size, with the lone exception of the European grayling *Thymallus thymallus*, whose genome has been subjected to an additional round of whole duplication compared with other Actinopterygii approximately 80 Mya (Sävilammi et al. 2019). These fractions of

pervasively transcribed regions were roughly 8 times lower than lungfish. Even though an increased amount of transcribed intergenic genomic sequence was also noted in the axolotl, this fraction was inferior by approximately 2.5 times compared with lungfish (fig. 1b, main text, table S5).

These observations were further supported by the contribution provided by such regions to the total transcriptional efforts in multiple tissues, which can be interpreted as a proxy for the energy investment of an organism towards pervasive transcription. The data collected about the expression of pervasively transcribed mRNAs clearly identified the lungfish as an outlier compared to all other species, including axolotl, where no more than 6.39% of all transcriptional effort was linked with this class of mRNAs (fig. 1c, main text, table S6). On average, despite the presence of a few outlier tissues and the slightly higher expression observed in *T. thymallus*, pervasive transcription only accounted for 3.06% (with a standard deviation of 2.34%) in bony fishes. Pervasive transcription was on the other hand highly prevalent in all lungfish tissues, with little variations between the two analyzed individuals, ranging from 24.30% in the liver of the first individual to 38.68% in the testis of the second individual. Overall, the testis was the tissue where pervasive transcription was more significant, which is consistent with the expected relaxed state of chromatin during spermatogenesis, which may increase its accessibility by the RNA polymerase II complex and accessory factors regulating transcription. Widespread intergenic transcription was less prominent in liver, whereas lung and brain showed an intermediate level of activity.

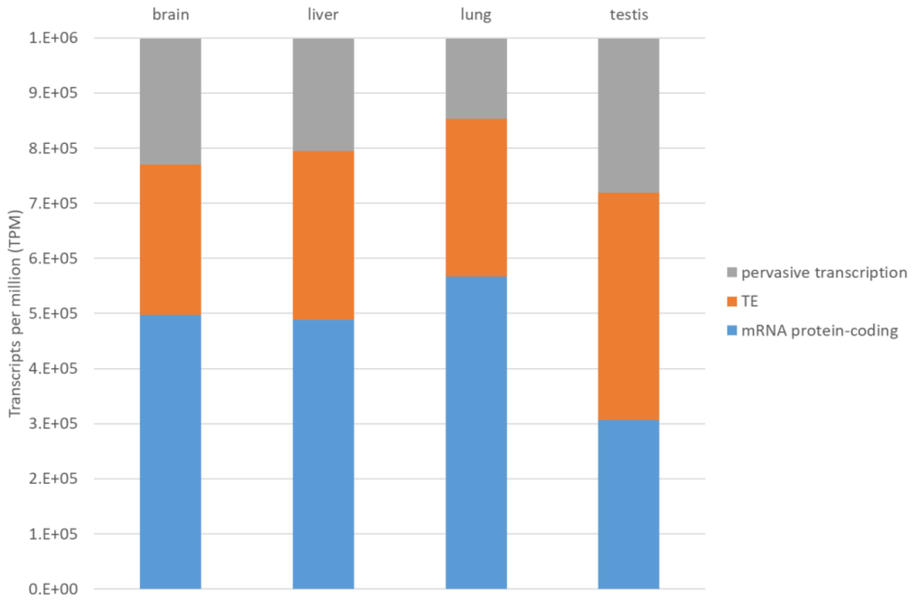

Figure S2. Total transcripts per million (TPM) of three main transcript categories in the *de novo* assembly, separated by tissue.

Table S5. Total size of the genomic regions subjected to intergenic pervasive transcription across different species and total fraction of the genome subjected to pervasive transcription (%), based on detected primary transcripts (i.e. by including introns).

species	assembled genome size	primary total pervasive size	primary pervasive proportion (%)
G. morhua	669,966,409	1,843,128	0.275
O. latipes	734,057,086	1,575,119	0.215
P. hypophthalmus	758,973,678	3,181,671	0.419
E. lucius	918,748,614	2,888,138	0.314
L. oculatus	945,878,036	4,398,397	0.465
P. fluviatilis	951,345,774	5,893,838	0.620
A. anguilla	979,045,926	3,885,796	0.397
T. thymallus	146,851,9221	19,922,580	1.357
S. trutta	2,371,880,186	13,492,506	0.569
A. mexicanum	28,206,906,136	469,750,929	1.665
N. forsteri	34,557,648,077	1,423,524,507	4.119

Table S6. Total contribution of pervasively transcribed intergenic regions to the global transcriptional effort in different tissues from multiple species. Gonads may refer either to the testis or to the ovary, depending on available data (see fig. 1c main text).

species	gonad	testis	ovary	muscle	lung	liver	kidney	intestine	heart	gills	embryo	cloacal glands	brain	bones
G. morhua		0.72928	0.43594	0.63267		2.15505	1.08730	1.69812	2.66852	3.37187	1.46186		3.18549	2.86295
O. latipes		2.09649	0.56929	0.53001		0.85087	1.26444	0.77612	1.65616	1.26105	1.00080		2.06287	1.09425
P. hypophthalmus		1.89776	2.06347	1.70824		2.00540	3.71246	3.41656	7.65047	3.03238	2.68048		4.86340	3.16821
E. lucius		6.31016	0.75197	2.66814		2.69259	1.90256	1.55633	1.64002	1.80856	0.94543		2.22028	1.44979
L. oculatus		2.86996	6.26903	3.20736		3.07154	2.13827	2.61353	3.38528	2.67230	1.89327		4.25334	3.06224
P. fluviatilis		1.52950	0.73149	1.88941		3.40406	2.03443	2.67971	3.30772	2.81026	3.44301		4.13730	3.19981
A. anguilla		1.57401	1.19006	1.75305		1.92266	2.53838	2.33083	1.91734	1.69504			3.23703	1.71217
T. thymallus		7.66690	3.99674	5.82503		8.37166	7.46513	8.30899	12.00762	7.35266	10.84386		10.28402	6.45929
S. trutta		2.73052	1.25829	1.30918		7.61352	2.49289	3.10417	4.38704	2.88818	1.38072		4.03335	1.92943
A. mexicanum	0.72988				0.52458	0.87048						6.38656		0.99468
	0.40746				0.53430	0.59098								1.00165
					0.64986	0.64852								1.05302
					0.52788	0.68726								
N. forsteri (indiv. 1)		37.25789			29.90884	24.30386							30.14285	
N. forsteri (indiv. 2)		38.68036			29.90535	24.43120							30.09291	
N. forsteri (PRJNA645042)	22.13765					20.14952							27.96540	

2.2 Relaxation of selection in lungfish and private nonsynonymous changes

Figure S3 shows the number of significant genes in CODEML and RELAX analysis. The first analysis was carried out with the aim of detecting any selective relaxation present in lungfish (foreground or test) relative to the rest of the species (SR1, fig. 2b, main text, and fig. S3). Out of the initial 1,790 genes, CODEML identified 611 genes for which a model with two different estimated ω for the two partitions was favored, compared to the null model with a single ω estimated for the entire topology (FDR < 0.01). For 601 of these 611 genes, the foreground ω resulted greater than that of the rest of the tree. This result suggests that 33.6% of the BUSCO genes analyzed showed a faster evolutionary rate in the lungfish branches ($\omega fg > \omega bg$), indicating either relaxation or positive selection. For the remaining 10 genes (0.6%) CODEML suggested higher conservation in the lungfish branches ($\omega fg < \omega bg$; fig. S3).

We further verified whether the pattern of relaxed selection was consistent in all or most of the branches, or if different genes were under relaxed selection in different evolutionary lineages. Most of the candidate genes for relaxed selection are found when assuming a different selective regime in the lungfish clade (Lung), and not its common ancestor (LNP), compared to the rest of the phylogeny (table S7, figure S4a). Accordingly, most of the genes under relaxed selection at the clade level are accumulating non synonymous substitutions in specific branches (see permutation tests in table S7), suggesting that relaxation was not gene specific but rather lineage specific. This reinforces the hypothesis that purifying selection is not efficient across the whole clade, resulting in independent and random accumulation of slightly deleterious mutations in each lineage. It is worth noting that these different patterns of selective regimes were identified despite the slower evolutionary rate in the lungfish branches than in the rest of the tree, affecting both nonsynonymous and synonymous sites (fig. S4b and S4c).

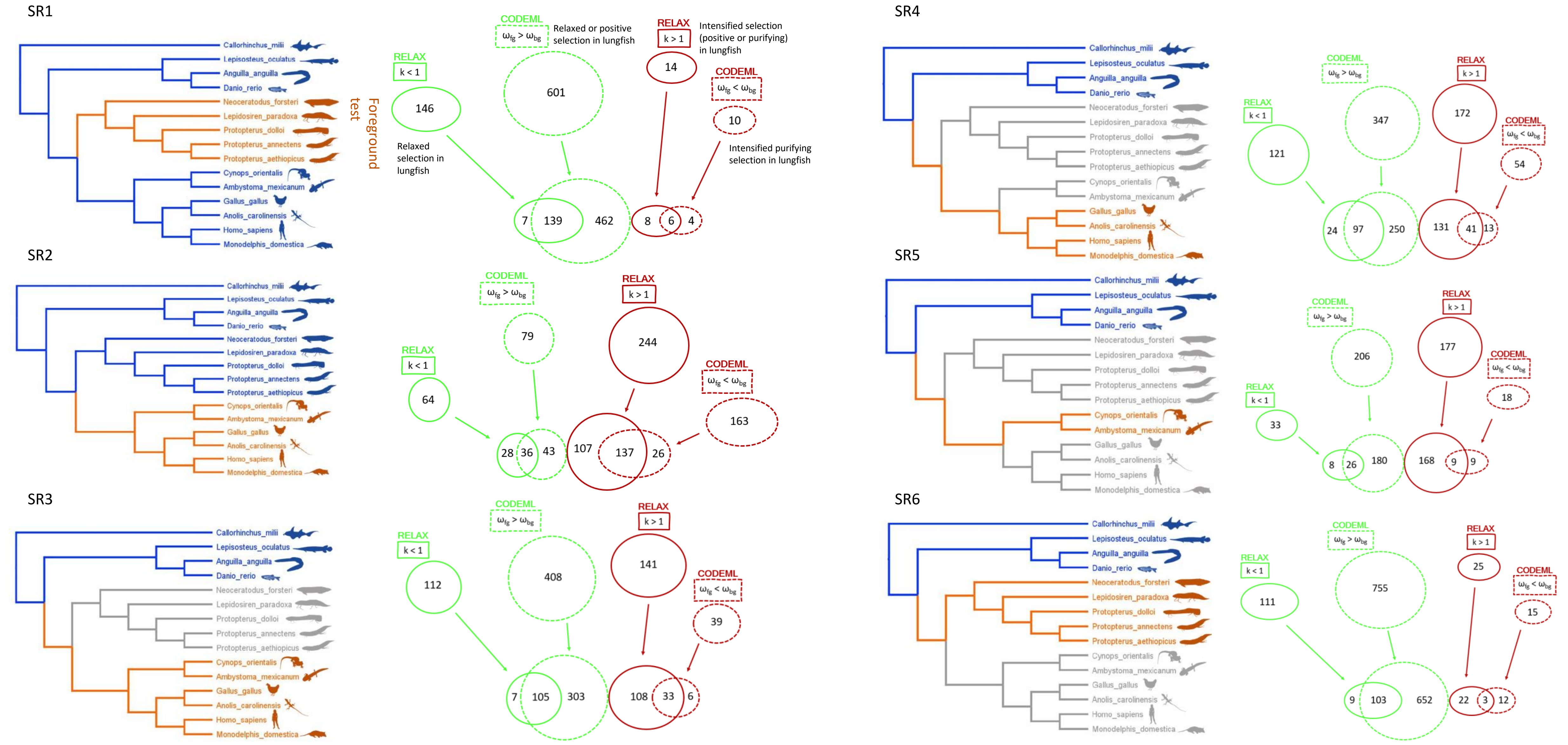

Figure S3. Detailed results of CODEML and RELAX analyses run starting from six different tree partitions (SR1-SR6). Blue branches: background/reference; orange branches: foreground/test; grey branches: excluded from the analysis. Dataset: 1,790 orthologous genes (or subsets). Venn diagrams show the number of genes for which analysis yielded significant results for CODEML and/or RELAX. Green: relaxed genes (RELAX, k<1, solid line) and/or genes with increased ω in the foreground (CODEML, ωfg > ωbg, discontinuous line); red: genes under intensified selection (RELAX, k>1, solid line) and/or genes under purifying selection (CODEML, ωfg < ωbg, discontinuous line).

Table S7. Candidate genes for relaxed selection across the lungfish phylogeny.

	**Candidate relaxed genes** [b]		**ω background branches** [c]		**ω foreground branch(es)** [c]	
**Foreground** [a]	***n***	***f***	**mean**	**(SD)**	**mean**	**(SD)**
**LNP & Lung**	388	0.65	0.082	(0.042)	0.128 (0.069); 0.207 (0.675)	
**(LNP+Lung)**	601	0.98	0.082	(0.042)	0.122	(0.064)
**LNP**	23	0.74	0.089	(0.045)	0.219	(0.720)
**Lung**	595	0.99	0.083	(0.042)	0.128	(0.069)
**Lpar**	167^{**}	0.99	0.087	(0.043)	0.259	(0.573)
**Nfor**	66^{**}	0.96	0.087	(0.044)	0.424	(1.044)
**Paet**	6^{*}	0.75	0.088	(0.044)	0.805	(1.557)
**Pann**	8^{*}	0.80	0.088	(0.044)	1.213	(1.824)
**Pdol**	32^{**}	0.94	0.088	(0.044)	1.020	(1.681)
**LP**	76^{**}	0.95	0.087	(0.044)	0.250	(0.736)
**P_2**	22^{**}	1.00	0.088	(0.044)	1.065	(1.712)
**P_3**	46^{**}	0.96	0.087	(0.043)	0.304	(0.840)
**$Lung_{any}$**	321	0.98	--		--	
**$LNP\text{-}or\text{-}Lung_{any}$**	335	0.97				
**$Lung\text{-}and\text{-}Lung_{any}$**	272	0.99	--		--	
**LNP_{only}**	20	0.70	--		--	

[a] Branch (or clade) set as foreground in the branch-test of CODEML: **LNP & Lung** = common ancestor of Lung and Lung had two independent selective pressures (candidate relaxed genes correspond to those that were detected as having a selective pressure (ω) significantly larger in both LNP and Lung; ω mean values for the two sets of branches are reported); **(LNP+Lung)** = common ancestor of Lung and Lung; **Lung** = lungfish clade; **LNP** = common ancestor of Lung; **Lpar** = terminal branch to *L. paradoxa*; **Nfor** = terminal branch to *N. forsteri*; **Paet** = terminal branch to *P. aethiopicus*; **Pann** = terminal branch to *P. annectens*; **Pdol** = terminal branch to *P. dolloi*; **LP** = common ancestor of *L. paradoxa* and the *Protopterus* clade; **P_2** = (*P. aethiopicus*, *P. annectens*) ancestor; **P_3** = *Protopterus* ancestor; **$Lung_{any}$** = in at least a branch within Lung; **$LNP\text{-}or\text{-}Lung_{any}$** = in LNP or at least a branch within Lung; **$Lung\text{-}and\text{-}Lung_{any}$** = in Lung and at least a branch within Lung; **LNP_{only}** = in LNP but not in Lung.

[b] Number of genes that have a selective pressure in the foreground branch(es) significantly larger than in the background branches (ωfg > ωbg; FDR < 0.01) according to the branch-test in CODEML. The fraction *f* refers to the ratio between this number (n) and the total number of genes that have a significantly different (either larger or lower) selective pressure in the foreground branch(es) than in the background branches.

[c] Mean (Standard Deviation) selective pressure intensity (ω) estimated across all 1790 genes, assuming two distinct values, one for foreground and one for the background branches.

*/** = the overlap between the candidate genes identified in the specific foreground branch and in the Lung clade is significantly larger than expected (100,000 permutations; * $P < 0.05$, **$P < 0.000001$).

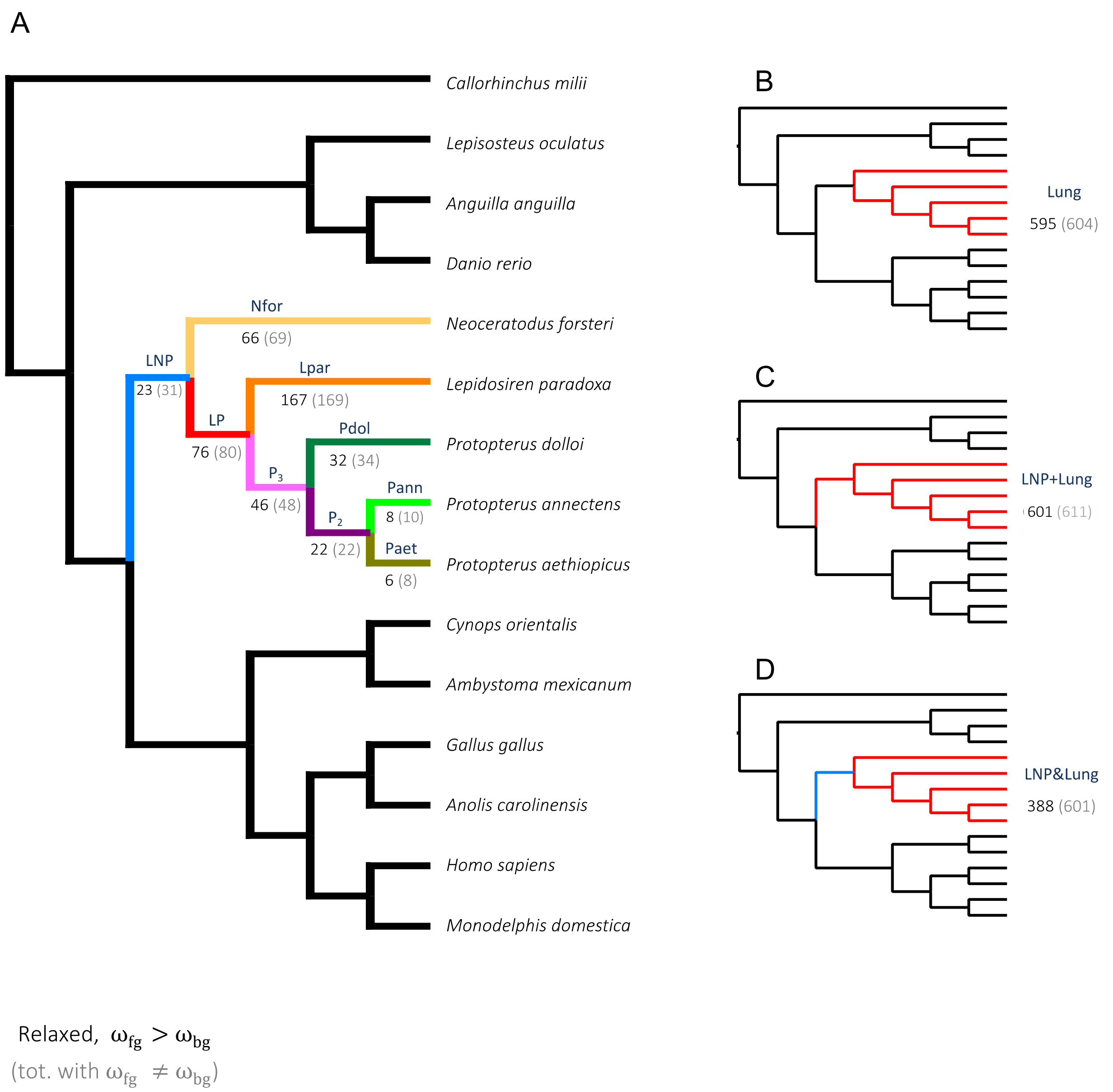

Figure S4a. Candidate genes for relaxed selection in lungfish. The number of genes detected as candidates for relaxed selection (FDR < 0.01) is shown for different configurations of foreground (in colour) and background branches (in black), which were assumed to be under distinct selective pressures (ω_{fg} and ω_{bg}, respectively). A) each terminal and internal branch was tested separately as foreground branch; B) the lungfish clade was considered as foreground; C) both the lungfish clade and its ancestor were considered as foreground; C) three ω values are assumed, one for the lungfish clade (ω_{fg1}), one for its ancestor (ω_{fg2}) and one for the rest of the phylogeny (ω_{bg}); genes were classified as candidates if $\omega_{fg1} > \omega_{bg}$ and $\omega_{fg2} > \omega_{bg}$. Branch codes are as in table S7.

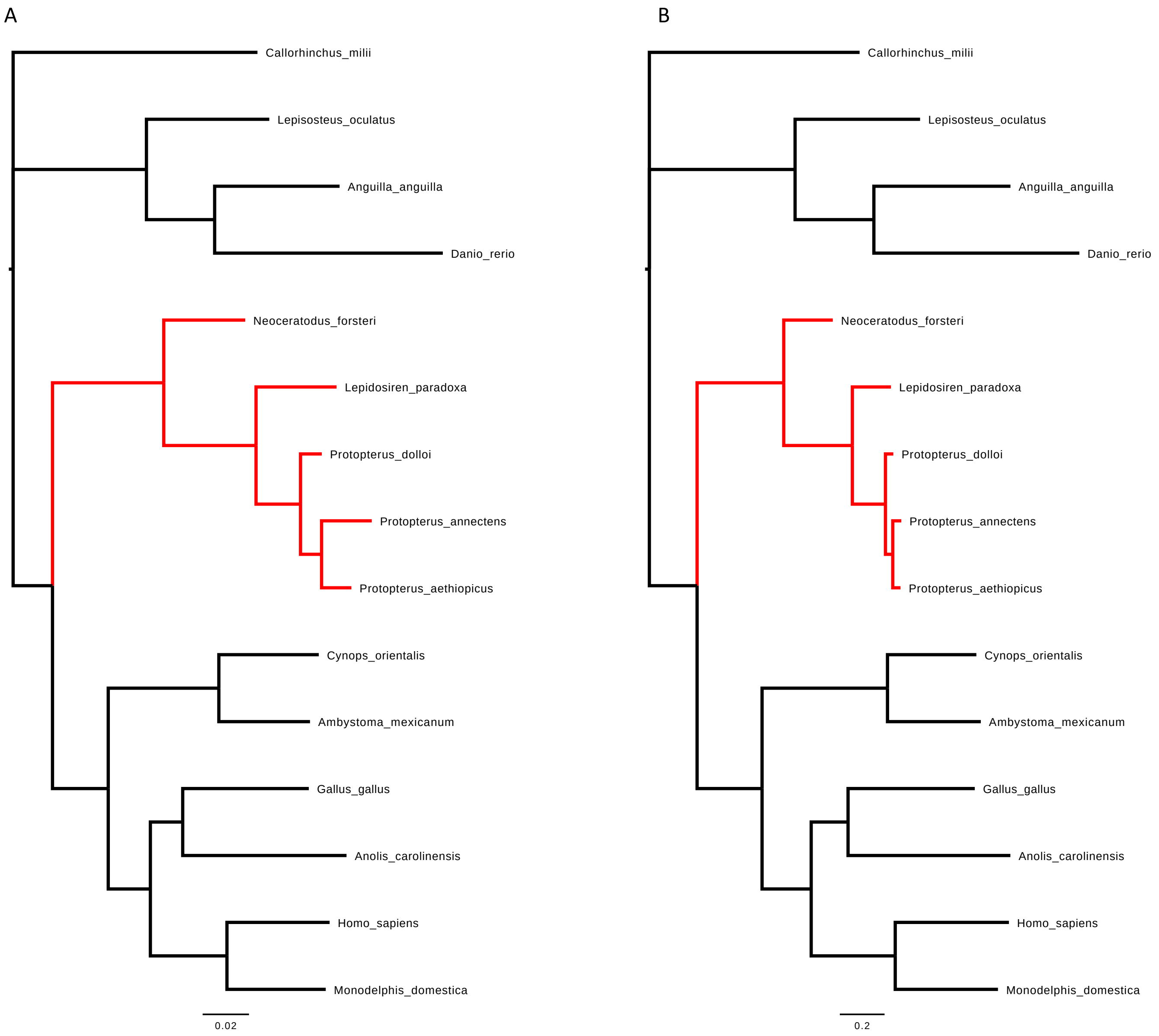

Figure S4b. Phylogenetic trees with topology and branch lengths corresponding to the average dN (panel A) and dS (panel B) values estimated using the two-ratio branch model in CODEML. The topology corresponds to the unrooted tree used to estimate substitution rates across the phylogeny. The lungfish clade (in red) was set as foreground branch and the rest of the phylogeny as background branches.

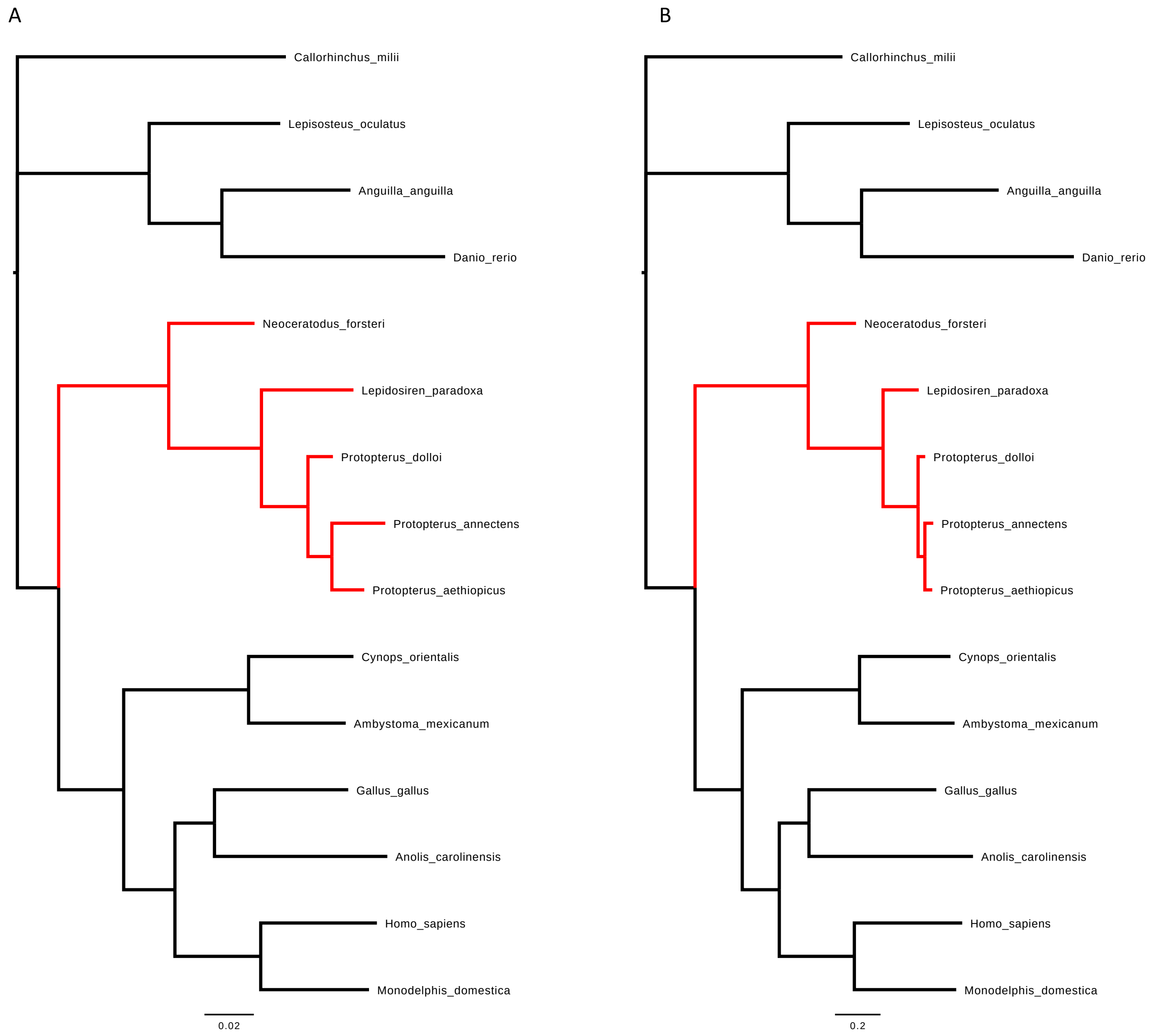

Figure S4c. Phylogenetic trees with branch lengths corresponding to the average dN (panel A) and dS (panel B) values estimated using the free-ratio model in CODEML. The topology corresponds to the unrooted tree used to estimate substitution rates across the phylogeny. The lungfish clade is highlighted in red. dS values were capped to 1.

The same 1,790 genes were analyzed with RELAX algorithm in two independent runs. After removing those genes that failed to produce an output in at least one of the two runs (n=87), 146 genes (8.9%) were identified as relaxed (k<1) and 14 genes (0.8%) under intensified selection (k>1) (fig. S3).

Comparing the results of CODEML and RELAX, 139 genes showed a consistent signal of relaxed selection in lungfish, while 6 genes consistently showed signatures of higher conservation in these branches (fig. S3, fig. 2b main text). When tetrapods instead of lungfish were compared with the rest of the tree (SR2), we observed an opposite trend. In particular, the number of relaxed genes appeared drastically reduced (CODEML $\omega fg > \omega bg$ intersection RELAX k<1 n=36), and the number of constrained genes increased (CODEML $\omega fg < \omega bg$ intersection RELAX k>1 n=137) (fig. S3). However, since the presence of the "relaxed" lungfish clade in the background/reference group may increase the signal of conservation in tetrapods, here representing the foreground/test, the same analyses were performed excluding the lungfish clade (SR3). In this case, a tetrapod-specific relaxation signal was detected for 105 genes (CODEML $\omega fg > \omega bg$ intersection RELAX k<1), while 33 genes resulted more constrained (CODEML $\omega fg < \omega bg$ intersection RELAX k>1) (fig S3). Although the number of relaxed genes in tetrapods increased when lungfish were excluded from the analysis (SR2=36 vs SR3=105), the relaxation signal remained significantly higher in lungfish than in tetrapods (chi-square test $p < 0.05$). Likewise, even though the estimated number of constrained genes in tetrapods decreased after removing lungfish from the analysis (SR2=137 vs SR3=33), constrained genes remained significantly underrepresented in lungfish than in tetrapods (chi-square test $p < 0.001$). By comparing SR3 and SR4, the effect of the giant urodele genomes in the foreground/test appeared negligible, while SR5 showed that the two urodeles alone in the foreground/test behave as the other tetrapods. Finally, SR6 showed that the lungfish maintained their relaxed signature when compared to the fish alone (fig. S3, fig. 2b main text).

Under strong selection relaxation, relaxed genes are expected to be enriched for deleterious mutations, and thus relaxed phylogenetic lineages are expected to show an excess of radical amino acid changes at conserved protein positions compared to the rest of the tree. To whether this was the case for the lungfish lineages, we translated the aligned coding sequences used for CODEML and RELAX analyses into their respective amino acid sequences, and we identified, in each of the 15 terminal branches of the tree in fig. 2a (main text), the positions where 14 lineages share the same amino acid, while a single lineage carries an alternative residue. These private amino acid changes were then classified into either radical or conservative according to the Conservative-Radical Index (CRI, Sharbrough et al. 2018). With the exception of *P. annectens*, the other four lungfish species show similar trends to the rest of the tree, suggesting that mutations with large selective coefficients are purged by purifying selection in relaxed phylogenetic lineages (**fig. S5**).

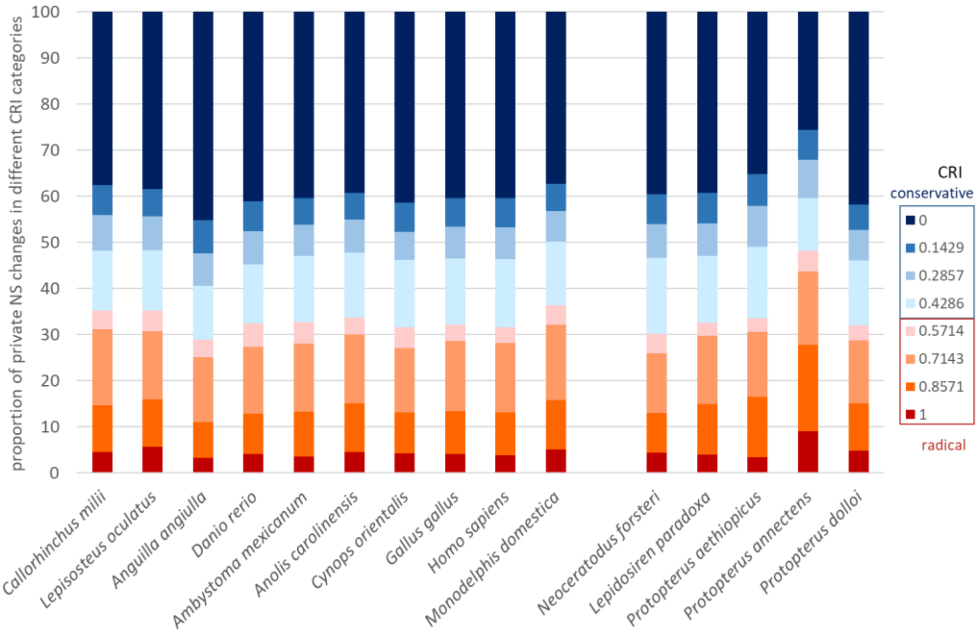

Figure S5. Proportion of private amino acid changes classified as radical (CRI > 0.5) or conservative (CRI<0.5) according to the overall index of the degree of amino acid change (CRI) developed by Sharbrough et al. (2018).

2.3 GO enrichment of genes under different evolutionary rates.

We found ten significantly enriched GO biological process or molecular function terms in the candidate genes for relaxation of selection suggested by both CODEML and RELAX, mostly related to protein turnover (table S8).

Table S8. GO enriched in the 139 genes identified as relaxed by CODEML and RELAX in lungfish compared with the rest of the phylogeny (FDR < 0.05 and Obs - Exp > 2).

GO ID	Obs-Exp	FDR	Description	Class
GO:0036459	3.912	0.0015	cysteine-type deubiquitinase activity	molecular_function
GO:0016567	5.435	0.0030	protein ubiquitination	biological_process
GO:0030334	2.456	0.0038	regulation of cell migration	biological_process
GO:0004843	3.679	0.0049	thiol-dependent deubiquitinase	molecular_function
GO:0031625	4.669	0.0064	ubiquitin protein ligase binding	molecular_function
GO:0043130	2.378	0.0070	ubiquitin binding	molecular_function
GO:0005770	2.912	0.0113	late endosome	cellular_component
GO:0034644	2.301	0.0117	cellular response to UV	biological_process
GO:0008333	2.223	0.0179	endosome to lysosome transport	biological_process
GO:0010008	2.145	0.0187	endosome membrane	cellular_component

Six genes turned out to be more constrained in lungfish than in the rest of the tree. These loci code for KIF1-binding protein (IPR022083), Mannose-6-phosphate isomerase (IPR016305), probable ATP-dependent DNA helicase HFM1 (IPR014001), ubiquitin carboxyl-terminal hydrolase 7 (IPR008974), podocin (IPR001107) and structural maintenance of chromosomes protein 5 (IPR027131).

1. Supplementary references

Alföldi J, Di Palma F, Grabherr M, Williams C, Kong L, Mauceli E, Russell P, Lowe CB, Glor RE, Jaffe JD, et al. 2011. The genome of the green anole lizard and a comparative analysis with birds and mammals. *Nature* 477:587–591.

Altschul SF, Gish W, Miller W, Myers EW, Lipman DJ. 1990. Basic local alignment search tool. *J. Mol. Biol.* 215:403–410.

Andrews S. 2010. FastQC: a quality control tool for high throughput sequence data. Available from: http://www.bioinformatics.babraham.ac.uk/projects/fastqc

Arnason U, Gullberg A, Janke A, Joss J, Elmerot C. 2004. Mitogenomic analyses of deep gnathostome divergences: a fish is a fish. *Gene* 333:61–70.

Ashburner M, Ball CA, Blake JA, Botstein D, Butler H, Cherry JM, Davis AP, Dolinski K, Dwight SS, Eppig JT, et al. 2000. Gene ontology: tool for the unification of biology. The Gene Ontology Consortium. *Nat. Genet.* 25:25–29.

Bankevich A, Nurk S, Antipov D, Gurevich AA, Dvorkin M, Kulikov AS, Lesin VM, Nikolenko SI, Pham S, Prjibelski AD, et al. 2012. SPAdes: A New Genome Assembly Algorithm and Its Applications to Single-Cell Sequencing. *J Comput Biol* 19:455–477.

Benjamini Y, Hochberg Y. 1995. Controlling the False Discovery Rate: A Practical and Powerful Approach to Multiple Testing. *Journal of the Royal Statistical Society: Series B (Methodological)* 57:289–300.

Biscotti MA, Carducci F, Barucca M, Gerdol M, Pallavicini A, Schartl M, Canapa A, Adolfi MC. 2020. The transcriptome of the newt *Cynops orientalis* provides new insights into evolution and function of sexual gene networks in sarcopterygians. *Sci Rep* 10:5445.

Biscotti MA, Gerdol M, Canapa A, Forconi M, Olmo E, Pallavicini A, Barucca M, Schartl M. 2016. The Lungfish Transcriptome: A Glimpse into Molecular Evolution Events at the Transition from Water to Land. *Sci Rep* 6:21571.

Braasch I, Gehrke AR, Smith JJ, Kawasaki K, Manousaki T, Pasquier J, Amores A, Desvignes T, Batzel P, Catchen J, et al. 2016. The spotted gar genome illuminates vertebrate evolution and facilitates human-teleost comparisons. *Nat Genet* 48:427–437.

Chen S, Zhou Y, Chen Y, Gu J. 2018. fastp: an ultra-fast all-in-one FASTQ preprocessor. *Bioinformatics* 34:i884–i890.

Criscuolo A, Gribaldo S. 2010. BMGE (Block Mapping and Gathering with Entropy): a new software for selection of phylogenetic informative regions from multiple sequence alignments. *BMC Evolutionary Biology* 10:210.

Danecek P, Bonfield JK, Liddle J, Marshall J, Ohan V, Pollard MO, Whitwham A, Keane T, McCarthy SA, Davies RM, et al. 2021. Twelve years of SAMtools and BCFtools. *Gigascience* 10:giab008.

Dobin A, Davis CA, Schlesinger F, Drenkow J, Zaleski C, Jha S, Batut P, Chaisson M, Gingeras TR. 2013. STAR: ultrafast universal RNA-seq aligner. *Bioinformatics* 29:15–21.

Fallon SJ, McDougall AJ, Espinoza T, Roberts DT, Brooks S, Kind PK, Kennard MJ, Bond N, Marshall SM, Schmidt D, et al. 2019. Age structure of the Australian lungfish (Neoceratodus forsteri). *PLoS One* 14:e0210168.

Finn RD, Clements J, Eddy SR. 2011. HMMER web server: interactive sequence similarity searching. *Nucl. Acids Res.* 39:W29–W37.

Grabherr MG, Haas BJ, Yassour M, Levin JZ, Thompson DA, Amit I, Adiconis X, Fan L, Raychowdhury R, Zeng Q, et al. 2011. Full-length transcriptome assembly from RNA-Seq data without a reference genome. *Nat Biotech* 29:644–652.

Hillier LW, Miller W, Birney E, Warren W, Hardison RC, Ponting CP, Bork P, Burt DW, Groenen MAM, Delany ME, et al. 2004. Sequence and comparative analysis of the chicken genome provide unique perspectives on vertebrate evolution. *Nature* 432:695–716.

Hoang DT, Chernomor O, von Haeseler A, Minh BQ, Vinh LS. 2018. UFBoot2: Improving the Ultrafast Bootstrap Approximation. *Mol Biol Evol* 35:518–522.

Howe K, Clark MD, Torroja CF, Torrance J, Berthelot C, Muffato M, Collins JE, Humphray S, McLaren K, Matthews L, et al. 2013. The zebrafish reference genome sequence and its relationship to the human genome. *Nature* 496:498–503.

Kalyaanamoorthy S, Minh BQ, Wong TKF, von Haeseler A, Jermiin LS. 2017. ModelFinder: fast model selection for accurate phylogenetic estimates. *Nat Methods* 14:587–589.

Kosakovsky Pond SL, Murrell B, Fourment M, Frost SDW, Delport W, Scheffler K. 2011. A Random Effects Branch-Site Model for Detecting Episodic Diversifying Selection. *Molecular Biology and Evolution* 28:3033–3043.

Köster J, Rahmann S. 2018. Snakemake—a scalable bioinformatics workflow engine. *Bioinformatics* 34:3600.

Kriventseva EV, Kuznetsov D, Tegenfeldt F, Manni M, Dias R, Simão FA, Zdobnov EM. 2019. OrthoDB v10: sampling the diversity of animal, plant, fungal, protist, bacterial and viral genomes for evolutionary and functional annotations of orthologs. *Nucleic Acids Research* 47:D807–D811.

Kriventseva EV, Rahman N, Espinosa O, Zdobnov EM. 2008. OrthoDB: the hierarchical catalog of eukaryotic orthologs. *Nucleic Acids Res.* 36:D271-275.

Letunic I, Bork P. 2019. Interactive Tree Of Life (iTOL) v4: recent updates and new developments. *Nucleic Acids Res* 47:W256–W259.

MacManes MD. 2018. The Oyster River Protocol: a multi-assembler and kmer approach for de novo transcriptome assembly. *PeerJ* 6:e5428.

Meyer A, Schloissnig S, Franchini P, Du K, Woltering JM, Irisarri I, Wong WY, Nowoshilow S, Kneitz S, Kawaguchi A, et al. 2021. Giant lungfish genome elucidates the conquest of land by vertebrates. *Nature* 590:284–289.

Mikkelsen TS, Wakefield MJ, Aken B, Amemiya CT, Chang JL, Duke S, Garber M, Gentles AJ, Goodstadt L, Heger A, et al. 2007. Genome of the marsupial *Monodelphis domestica* reveals innovation in non-coding sequences. *Nature* 447:167–177.

Neph S, Kuehn MS, Reynolds AP, Haugen E, Thurman RE, Johnson AK, Rynes E, Maurano MT, Vierstra J, Thomas S, et al. 2012. BEDOPS: high-performance genomic feature operations. *Bioinformatics* 28:1919–1920.

Nguyen L-T, Schmidt HA, von Haeseler A, Minh BQ. 2015. IQ-TREE: a fast and effective stochastic algorithm for estimating maximum-likelihood phylogenies. *Mol Biol Evol* 32:268–274.

Nogueira AF, Costa CM, Lorena J, Moreira RN, Frota-Lima GN, Furtado C, Robinson M, Amemiya CT, Darnet S, Schneider I. 2016. Tetrapod limb and sarcopterygian fin regeneration share a core genetic programme. *Nat Commun* 7:13364.

Patro R, Duggal G, Love MI, Irizarry RA, Kingsford C. 2017. Salmon: fast and bias-aware quantification of transcript expression using dual-phase inference. *Nat Methods* 14:417–419.

Price AL, Jones NC, Pevzner PA. 2005. *De novo* identification of repeat families in large genomes. *Bioinformatics* 21:i351–i358.

Punta M, Coggill PC, Eberhardt RY, Mistry J, Tate J, Boursnell C, Pang N, Forslund K, Ceric G, Clements J, et al. 2012. The Pfam protein families database. *Nucleic Acids Research* 40:D290–D301.

Quinlan AR, Hall IM. 2010. BEDTools: a flexible suite of utilities for comparing genomic features. *Bioinformatics* 26:841–842.

Ranwez V, Harispe S, Delsuc F, Douzery EJP. 2011. MACSE: Multiple Alignment of Coding SEquences Accounting for Frameshifts and Stop Codons. *PLOS ONE* 6:e22594.

Rhie A, McCarthy SA, Fedrigo O, Damas J, Formenti G, Koren S, Uliano-Silva M, Chow W, Fungtammasan A, Kim J, et al. 2021. Towards complete and error-free genome assemblies of all vertebrate species. *Nature* 592:737–746.

Riehl K, Riccio C, Miska EA, Hemberg M. 2022. TransposonUltimate: software for transposon classification, annotation and detection. *Nucleic Acids Research* 50:e64.

Robertson G, Schein J, Chiu R, Corbett R, Field M, Jackman SD, Mungall K, Lee S, Okada HM, Qian JQ, et al. 2010. *De novo* assembly and analysis of RNA-seq data. *Nature Methods* 7:909–912.

Sävilammi T, Primmer CR, Varadharajan S, Guyomard R, Guiguen Y, Sandve SR, Vøllestad LA, Papakostas S, Lien S. 2019. The Chromosome-Level Genome Assembly of European Grayling Reveals Aspects of a Unique Genome Evolution Process Within Salmonids. *G3 (Bethesda)* 9:1283–1294.

Sharbrough J, Luse M, Boore JL, Logsdon JM, Neiman M. 2018. Radical amino acid mutations persist longer in the absence of sex. *Evolution* 72:808–824.

Simão FA, Waterhouse RM, Ioannidis P, Kriventseva EV, Zdobnov EM. 2015. BUSCO: assessing genome assembly and annotation completeness with single-copy orthologs. *Bioinformatics* 31:3210–3212.

Smit A. 2015. Smit, AFA, Hubley, R & Green, P. RepeatMasker Open-4.0.

Smith JJ, Timoshevskaya N, Timoshevskiy VA, Keinath MC, Hardy D, Voss SR. 2019. A chromosome-scale assembly of the axolotl genome. *Genome Res* 29:317–324.

Takezaki N, Nishihara H. 2017. Support for Lungfish as the Closest Relative of Tetrapods by Using Slowly Evolving Ray-Finned Fish as the Outgroup. *Genome Biol Evol* 9:93–101.

Venkatesh B, Lee AP, Ravi V, Maurya AK, Lian MM, Swann JB, Ohta Y, Flajnik MF, Sutoh Y, Kasahara M, et al. 2014. Elephant shark genome provides unique insights into gnathostome evolution. *Nature* 505:174–179.

Wertheim JO, Murrell B, Smith MD, Kosakovsky Pond SL, Scheffler K. 2015. RELAX: detecting relaxed selection in a phylogenetic framework. *Mol Biol Evol* 32:820–832.

Wright C, Rajpurohit A, Burke EE, Williams C, Collado-Torres L, Kimos M, Brandon NJ, Cross AJ, Jaffe AE, Weinberger DR, et al. 2019. Comprehensive assessment of multiple biases in small RNA sequencing reveals significant differences in the performance of widely used methods. *BMC Genomics* 20:513.

Yang Z. 1998. Likelihood ratio tests for detecting positive selection and application to primate lysozyme evolution. *Mol Biol Evol* 15:568–573.

Yang Z. 2007. PAML 4: phylogenetic analysis by maximum likelihood. *Mol Biol Evol* 24:1586–1591.

Zardoya R, Meyer A. 1996. Evolutionary relationships of the coelacanth, lungfishes, and tetrapods based on the 28S ribosomal RNA gene. *Proc. Natl. Acad. Sci. U.S.A.* 93:5449–5454.
